# Supplementary material for: Conflict, healthcare and professional perseverance: A qualitative study in a remote hospital in an Anglophone Region of Cameroon
Source: PLOS Glob Public Health. 2022 Nov 29;2(11):e0001145. doi: 10.1371/journal.pgph.0001145 (PMC10021219; doi:10.1371/journal.pgph.0001145)
Supplement: S1 Table — (DOC) [file pgph.0001145.s001.doc]

FREQUENCIES VARIABLES=age maritalstatus
  /NTILES=4
  /STATISTICS=STDDEV MEAN
  /ORDER=ANALYSIS.


Frequencies


Notes	
Output Created	
Comments	
Input	Active Dataset	
	Filter	
	Weight	
	Split File	
	N of Rows in Working Data File	
Missing Value Handling	Definition of Missing	
	Cases Used	
Syntax	
Resources	Processor Time	
	Elapsed Time	

Notes	
Output Created	12-APR-2022 20:47:29	
Comments		
Input	Active Dataset	DataSet0	
	Filter	<none>	
	Weight	<none>	
	Split File	<none>	
	N of Rows in Working Data File	12	
Missing Value Handling	Definition of Missing	User-defined missing values are treated as missing.	
	Cases Used	Statistics are based on all cases with valid data.	
Syntax	FREQUENCIES VARIABLES=age maritalstatus
  /NTILES=4
  /STATISTICS=STDDEV MEAN
  /ORDER=ANALYSIS.	
Resources	Processor Time	00:00:00,00	
	Elapsed Time	00:00:00,02	


[DataSet0] 


Statistics	
	Participants age	
N	Valid	12	
	Missing	0	
Mean	33,67	
Std. Deviation	6,719	
Percentiles	25	27,75	
	50	32,50	
	75	37,25	

Statistics	
	marital status	
N	Valid	12	
	Missing	0	
Mean	,50	
Std. Deviation	,522	
Percentiles	25	,00	
	50	,50	
	75	1,00	


Frequency Table


Participants age	
	Frequency	Percent	
Valid	25	1	8,3	
	27	2	16,7	
	30	1	8,3	
	32	2	16,7	
	33	1	8,3	
	34	1	8,3	
	35	1	8,3	
	38	1	8,3	
	43	1	8,3	
	48	1	8,3	
	Total	12	100,0	

Participants age	
	Valid Percent	
Valid	25	8,3	
	27	16,7	
	30	8,3	
	32	16,7	
	33	8,3	
	34	8,3	
	35	8,3	
	38	8,3	
	43	8,3	
	48	8,3	
	Total	100,0	

Participants age	
	Cumulative Percent	
Valid	25	8,3	
	27	25,0	
	30	33,3	
	32	50,0	
	33	58,3	
	34	66,7	
	35	75,0	
	38	83,3	
	43	91,7	
	48	100,0	
	Total		


marital status	
	Frequency	Percent	
Valid	single	6	50,0	
	married	6	50,0	
	Total	12	100,0	

marital status	
	Valid Percent	
Valid	single	50,0	
	married	50,0	
	Total	100,0	

marital status	
	Cumulative Percent	
Valid	single	50,0	
	married	100,0	
	Total		

FREQUENCIES VARIABLES=age maritalstatus profession
  /NTILES=4
  /STATISTICS=STDDEV MEAN
  /ORDER=ANALYSIS.


Frequencies


Notes	
Output Created	
Comments	
Input	Active Dataset	
	Filter	
	Weight	
	Split File	
	N of Rows in Working Data File	
Missing Value Handling	Definition of Missing	
	Cases Used	
Syntax	
Resources	Processor Time	
	Elapsed Time	

Notes	
Output Created	12-APR-2022 20:48:46	
Comments		
Input	Active Dataset	DataSet0	
	Filter	<none>	
	Weight	<none>	
	Split File	<none>	
	N of Rows in Working Data File	12	
Missing Value Handling	Definition of Missing	User-defined missing values are treated as missing.	
	Cases Used	Statistics are based on all cases with valid data.	
Syntax	FREQUENCIES VARIABLES=age maritalstatus profession
  /NTILES=4
  /STATISTICS=STDDEV MEAN
  /ORDER=ANALYSIS.	
Resources	Processor Time	00:00:00,00	
	Elapsed Time	00:00:00,01	


[DataSet0] 


Statistics	
	Participants age	
N	Valid	12	
	Missing	0	
Mean	33,67	
Std. Deviation	6,719	
Percentiles	25	27,75	
	50	32,50	
	75	37,25	

Statistics	
	marital status	
N	Valid	12	
	Missing	0	
Mean	,50	
Std. Deviation	,522	
Percentiles	25	,00	
	50	,50	
	75	1,00	

Statistics	
	profession	
N	Valid	12	
	Missing	0	
Mean	,42	
Std. Deviation	,669	
Percentiles	25	,00	
	50	,00	
	75	1,00	


Frequency Table


Participants age	
	Frequency	Percent	
Valid	25	1	8,3	
	27	2	16,7	
	30	1	8,3	
	32	2	16,7	
	33	1	8,3	
	34	1	8,3	
	35	1	8,3	
	38	1	8,3	
	43	1	8,3	
	48	1	8,3	
	Total	12	100,0	

Participants age	
	Valid Percent	
Valid	25	8,3	
	27	16,7	
	30	8,3	
	32	16,7	
	33	8,3	
	34	8,3	
	35	8,3	
	38	8,3	
	43	8,3	
	48	8,3	
	Total	100,0	

Participants age	
	Cumulative Percent	
Valid	25	8,3	
	27	25,0	
	30	33,3	
	32	50,0	
	33	58,3	
	34	66,7	
	35	75,0	
	38	83,3	
	43	91,7	
	48	100,0	
	Total		


marital status	
	Frequency	Percent	
Valid	single	6	50,0	
	married	6	50,0	
	Total	12	100,0	

marital status	
	Valid Percent	
Valid	single	50,0	
	married	50,0	
	Total	100,0	

marital status	
	Cumulative Percent	
Valid	single	50,0	
	married	100,0	
	Total		


profession	
	Frequency	Percent	
Valid	nurse	8	66,7	
	doctor	3	25,0	
	labtech	1	8,3	
	Total	12	100,0	

profession	
	Valid Percent	
Valid	nurse	66,7	
	doctor	25,0	
	labtech	8,3	
	Total	100,0	

profession	
	Cumulative Percent	
Valid	nurse	66,7	
	doctor	91,7	
	labtech	100,0	
	Total		

FREQUENCIES VARIABLES=age maritalstatus profession sex
  /NTILES=4
  /STATISTICS=STDDEV MEAN
  /ORDER=ANALYSIS.


Frequencies


Notes	
Output Created	
Comments	
Input	Active Dataset	
	Filter	
	Weight	
	Split File	
	N of Rows in Working Data File	
Missing Value Handling	Definition of Missing	
	Cases Used	
Syntax	
Resources	Processor Time	
	Elapsed Time	

Notes	
Output Created	12-APR-2022 20:49:25	
Comments		
Input	Active Dataset	DataSet0	
	Filter	<none>	
	Weight	<none>	
	Split File	<none>	
	N of Rows in Working Data File	12	
Missing Value Handling	Definition of Missing	User-defined missing values are treated as missing.	
	Cases Used	Statistics are based on all cases with valid data.	
Syntax	FREQUENCIES VARIABLES=age maritalstatus profession sex
  /NTILES=4
  /STATISTICS=STDDEV MEAN
  /ORDER=ANALYSIS.	
Resources	Processor Time	00:00:00,00	
	Elapsed Time	00:00:00,03	


[DataSet0] 


Statistics	
	Participants age	
N	Valid	12	
	Missing	0	
Mean	33,67	
Std. Deviation	6,719	
Percentiles	25	27,75	
	50	32,50	
	75	37,25	

Statistics	
	marital status	
N	Valid	12	
	Missing	0	
Mean	,50	
Std. Deviation	,522	
Percentiles	25	,00	
	50	,50	
	75	1,00	

Statistics	
	profession	
N	Valid	12	
	Missing	0	
Mean	,42	
Std. Deviation	,669	
Percentiles	25	,00	
	50	,00	
	75	1,00	

Statistics	
	Gender	
N	Valid	12	
	Missing	0	
Mean		
Std. Deviation		
Percentiles	25		
	50		
	75		


Frequency Table


Participants age	
	Frequency	Percent	
Valid	25	1	8,3	
	27	2	16,7	
	30	1	8,3	
	32	2	16,7	
	33	1	8,3	
	34	1	8,3	
	35	1	8,3	
	38	1	8,3	
	43	1	8,3	
	48	1	8,3	
	Total	12	100,0	

Participants age	
	Valid Percent	
Valid	25	8,3	
	27	16,7	
	30	8,3	
	32	16,7	
	33	8,3	
	34	8,3	
	35	8,3	
	38	8,3	
	43	8,3	
	48	8,3	
	Total	100,0	

Participants age	
	Cumulative Percent	
Valid	25	8,3	
	27	25,0	
	30	33,3	
	32	50,0	
	33	58,3	
	34	66,7	
	35	75,0	
	38	83,3	
	43	91,7	
	48	100,0	
	Total		


marital status	
	Frequency	Percent	
Valid	single	6	50,0	
	married	6	50,0	
	Total	12	100,0	

marital status	
	Valid Percent	
Valid	single	50,0	
	married	50,0	
	Total	100,0	

marital status	
	Cumulative Percent	
Valid	single	50,0	
	married	100,0	
	Total		


profession	
	Frequency	Percent	
Valid	nurse	8	66,7	
	doctor	3	25,0	
	labtech	1	8,3	
	Total	12	100,0	

profession	
	Valid Percent	
Valid	nurse	66,7	
	doctor	25,0	
	labtech	8,3	
	Total	100,0	

profession	
	Cumulative Percent	
Valid	nurse	66,7	
	doctor	91,7	
	labtech	100,0	
	Total		


Gender	
	Frequency	Percent	
Valid	female	6	50,0	
	male	6	50,0	
	Total	12	100,0	

Gender	
	Valid Percent	
Valid	female	50,0	
	male	50,0	
	Total	100,0	

Gender	
	Cumulative Percent	
Valid	female	50,0	
	male	100,0	
	Total		
