# Supplementary material for: Conflict, healthcare and professional perseverance: A qualitative study in a remote hospital in an Anglophone Region of Cameroon
Source: PLOS Glob Public Health. 2022 Nov 29;2(11):e0001145. doi: 10.1371/journal.pgph.0001145 (PMC10021219; doi:10.1371/journal.pgph.0001145)
Supplement: S2 Table — (PDF) [file pgph.0001145.s002.pdf]

ID	Document
9:9	RESPONDENT 1- adult male nurse

Quotation Content

Yes, yes. Unfortunately one of our doctors was attacked although, not in the health facility. He travelled to Bamenda to run an errand for the health facility where he was attacked and the fingers cut off. So we suffered a lot in the health facility because the dexterity of his fingers was lost and he could not perform surgeries. It was not easy for us.

Comment

Functionally impairing a key personnel thus affecting effective healthcare delivery

Codes	Reference	Modified by
Physical assault of health workers	21 - 21	Juste Niba
