# Supplementary material for: Conflict, healthcare and professional perseverance: A qualitative study in a remote hospital in an Anglophone Region of Cameroon
Source: PLOS Glob Public Health. 2022 Nov 29;2(11):e0001145. doi: 10.1371/journal.pgph.0001145 (PMC10021219; doi:10.1371/journal.pgph.0001145)
Supplement: S3 Table — (PDF) [file pgph.0001145.s003.pdf]

ID	Document
11:6	FG discussion 1

Quotation Content

Yes, actually there is an incident we heard of in Mbengwi involving two nurses, a man and his wife shot by the armed forces (military)

Comment

Were they targetted because of their status as health personnel?

Codes	Reference	Modified by
health workers murdered	13 - 13	Juste Niba
