# Supplementary material for: Conflict, healthcare and professional perseverance: A qualitative study in a remote hospital in an Anglophone Region of Cameroon
Source: PLOS Glob Public Health. 2022 Nov 29;2(11):e0001145. doi: 10.1371/journal.pgph.0001145 (PMC10021219; doi:10.1371/journal.pgph.0001145)
Supplement: S4 Table — (PDF) [file pgph.0001145.s004.pdf]

| ID | Document |
| --- | --- |
| 9:18 | RESPONDENT 1- adult male nurse |
| 11:17 | FG discussion 1 |

**Quotation Content**

Firstly is the insecurity. Most patients will not come to the hospital at a time when the atmosphere is tense and others will not like to visit this health centers especially with people (armed forces) patrolling the streets

There are also some patients who leave from far distances to reach the hospital and because of the insecurity; it is difficult for them to move

**Comment**

Patients are afraid of leaving their homes to come to the hospital especialy those who live far away

| Codes | Reference | Modified by |
| --- | --- | --- |
| overall insecurity | 36 - 36 | Juste Niba |
| overall insecurity | 20 - 20 | Juste Niba |
