# Supplementary material for: Conflict, healthcare and professional perseverance: A qualitative study in a remote hospital in an Anglophone Region of Cameroon
Source: PLOS Glob Public Health. 2022 Nov 29;2(11):e0001145. doi: 10.1371/journal.pgph.0001145 (PMC10021219; doi:10.1371/journal.pgph.0001145)
Supplement: S5 Table — (PDF) [file pgph.0001145.s005.pdf]

ID	Document
10:8	RESPONDENT 2- adult female nurse
10:15	RESPONDENT 2- adult female nurse
10:18	RESPONDENT 2- adult female nurse

Quotation Content

they escape into the bushes with the mosquitoes there. So they are exposed to bites, poor feeding like just cocoyam and oil, children become malnourished, they contract malaria and after the lockdown, the cases start coming in multitudes.

And yes we are seeing a lot of hypertension now because of the stress. People have traumatic stress disorder from their past experiences

So you see why more people are hypertensive, with the poor diet, diabetes and everything is on the rise.

Comment

Codes	Reference	Modified by
Disease complications<br>Increase disease prevalence	8 - 8	Juste Niba
Increase disease prevalence	12 - 12	Juste Niba
Increase disease prevalence	12 - 12	Juste Niba
