# Supplementary material for: Conflict, healthcare and professional perseverance: A qualitative study in a remote hospital in an Anglophone Region of Cameroon
Source: PLOS Glob Public Health. 2022 Nov 29;2(11):e0001145. doi: 10.1371/journal.pgph.0001145 (PMC10021219; doi:10.1371/journal.pgph.0001145)
Supplement: S6 Table — (PDF) [file pgph.0001145.s006.pdf]

ID	Document
10:8	RESPONDENT 2-<br>adult female nurse
11:36	FG discussion 1

Quotation Content

they escape into the bushes with the mosquitoes there. So they are exposed to bites, poor feeding like just cocoyam and oil, children become malnourished, they contract malaria and after the lockdown, the cases start coming in multitudes.

most people are not able to reach the hospital, most women deliver at home. It is after weeks or a month of lockdown when the roads are accessible we start seeing critical cases and most of them die because they were not able to have access to health care

Comment

All because of prolongued road blocks

Codes	Reference	Modified by
Disease complications<br>Increase disease prevalence	8 - 8	Juste Niba
Delivery at home<br>Disease complications	39 - 39	Juste Niba
