# Supplementary material for: Conflict, healthcare and professional perseverance: A qualitative study in a remote hospital in an Anglophone Region of Cameroon
Source: PLOS Glob Public Health. 2022 Nov 29;2(11):e0001145. doi: 10.1371/journal.pgph.0001145 (PMC10021219; doi:10.1371/journal.pgph.0001145)
Supplement: S7 Table — (PDF) [file pgph.0001145.s007.pdf]

ID	Document
9:23	RESPONDENT 1- adult male nurse

Quotation Content

This is a place of refuge for many so during that time the health center is crowded with people and all the staff live here until the period is over. So these are some of the ways they support and encourage us and it is good.

Comment

Codes	Reference	Modified by
hospital a safe haven for all	41 - 41	Juste Niba
