# Supplementary material for: Conflict, healthcare and professional perseverance: A qualitative study in a remote hospital in an Anglophone Region of Cameroon
Source: PLOS Glob Public Health. 2022 Nov 29;2(11):e0001145. doi: 10.1371/journal.pgph.0001145 (PMC10021219; doi:10.1371/journal.pgph.0001145)
Supplement: S8 Table — (PDF) [file pgph.0001145.s008.pdf]

ID	Document
10:11	RESPONDENT 2-adult female nurse
10:41	RESPONDENT 2-adult female nurse

Quotation Content

This is the point I usually make when I'm appealing to some of these organizations. I always tell them the people cannot afford and if they can provide some free services it will really help the people a lot

There were so many children in the compound and thanks to some organizations who helped to buy food for the children

Comment

Codes	Reference	Modified by
Free aid	10 - 10	Juste Niba
Free aid	29 - 29	Juste Niba
