# Supplementary material for: Conflict, healthcare and professional perseverance: A qualitative study in a remote hospital in an Anglophone Region of Cameroon
Source: PLOS Glob Public Health. 2022 Nov 29;2(11):e0001145. doi: 10.1371/journal.pgph.0001145 (PMC10021219; doi:10.1371/journal.pgph.0001145)
Supplement: S9 Table — (PDF) [file pgph.0001145.s009.pdf]

| ID | Document |
| --- | --- |
| 9:17 | RESPONDENT 1- adult male nurse |
| 9:30 | RESPONDENT 1- adult male nurse |
| 10:20 | RESPONDENT 2- adult female nurse |

**Quotation Content**

There should be a cease fire between both parties then we are going to have peace. If that is done there will be peace and we can work freely within the communities and many patients will have access to medical care at any time

First thing I would say is that the government should do something about this situation. There should be a cease fire between both parties. You know when the elephant is fighting, what suffers is the grass

Whatever needs to be done must be done because it is a chaos. Pull out the military, solve the problem of the boys and solve the Anglophone crisis

**Comment**

This is not a solution put into action yet. It is just a suggested approach that could possibly solve the problem

| Codes | Reference | Modified by |
|---|---|---|
| Cease fire | 34 - 34 | Juste Niba |
| Cease fire | 48 - 48 | Juste Niba |
| Cease fire<br>government intervention | 14 - 14 | Juste Niba |
