# Supplementary material for: Conflict, healthcare and professional perseverance: A qualitative study in a remote hospital in an Anglophone Region of Cameroon
Source: PLOS Glob Public Health. 2022 Nov 29;2(11):e0001145. doi: 10.1371/journal.pgph.0001145 (PMC10021219; doi:10.1371/journal.pgph.0001145)
Supplement: S10 Table — (PDF) [file pgph.0001145.s010.pdf]

| ID | Document |
| --- | --- |
| 11:37 | FG discussion 1 |

**Quotation Content**

So maybe if community health workers are placed at the disposal of every community they will be able to reach them and administer baseline medications on time

### Comment

| Codes | Reference | Modified by |
|---|---|---|
| community health workers | 39 - 39 | Juste Niba |
