# Supplementary material for: Conflict, healthcare and professional perseverance: A qualitative study in a remote hospital in an Anglophone Region of Cameroon
Source: PLOS Glob Public Health. 2022 Nov 29;2(11):e0001145. doi: 10.1371/journal.pgph.0001145 (PMC10021219; doi:10.1371/journal.pgph.0001145)
Supplement: S11 Table — (PDF) [file pgph.0001145.s011.pdf]

ID	Document
9:16	RESPONDENT 1- adult male nurse
9:22	RESPONDENT 1- adult male nurse
10:25	RESPONDENT 2- adult female nurse

Quotation Content

But when I came back, we received encouragement and reassurance. Thank God the next day it was much calm, like I would have gone.

Firstly if things normalize, if there is a cease fire, I will not leave and more so I would have gone if not for the encouragement given to us by the administrator.

Ive been sent here on a divine mission, therefore I will always choose to be here because my reason is divine and not humanly

Comment

This respondent has a specific purpose (divine mission) for working

Codes	Reference	Modified by
Motivation to work	32 - 32	Juste Niba
Motivation to work	41 - 41	Juste Niba
Motivation to work	17 - 17	Juste Niba
