# Supplementary material for: Conflict, healthcare and professional perseverance: A qualitative study in a remote hospital in an Anglophone Region of Cameroon
Source: PLOS Glob Public Health. 2022 Nov 29;2(11):e0001145. doi: 10.1371/journal.pgph.0001145 (PMC10021219; doi:10.1371/journal.pgph.0001145)
Supplement: Attachment — Submitted filename: Response to Reviewers.docx [file pgph.0001145.s012.docx]

Juste Niba

12^th^ September 2022

Douala, Cameroon

The Editor,

PLOS Global Public Health

Dear Sir,

Rebuttal Letter

We recently submitted our manuscript entitled: Conflict, Healthcare and Professional Perseverance: A Qualitative study in an Anglophone Region in Cameroon. You have asked for a revision of the manuscript. Please find below the comments regarding the issues you wanted us to address. Our added comments appear in red.

Yours sincerely,

Juste Niba

Journal Requirements:

1. Please ensure that you refer to the main tables in your text as, if accepted, production will need these reference to link the reader to the tables.

Thank you for the remark. All tables have been referred in the main text where necessary.

Additional Editor Comments (if provided):

Dear Authors,

I would encourage you to revise and resubmit. Please address the concerns that the reviewers have on your paper. Looking forward to reading the resubmission.


[Note: HTML markup is below. Please do not edit.]

Reviewers' comments:

Reviewer's Responses to Questions

**Comments to the Author**

1. Does this manuscript meet PLOS Global Public Health’s [publication criteria](https://journals.plos.org/globalpublichealth/s/criteria-for-publication)? Is the manuscript technically sound, and do the data support the conclusions? The manuscript must describe methodologically and ethically rigorous research with conclusions that are appropriately drawn based on the data presented.

Reviewer #1: Partly

Reviewer #2: No

2. Has the statistical analysis been performed appropriately and rigorously?

Reviewer #1: Yes

Reviewer #2: No

3. Have the authors made all data underlying the findings in their manuscript fully available (please refer to the Data Availability Statement at the start of the manuscript PDF file)?

The [PLOS Data policy](https://journals.plos.org/globalpublichealth/s/data-availability) requires authors to make all data underlying the findings described in their manuscript fully available without restriction, with rare exception. The data should be provided as part of the manuscript or its supporting information, or deposited to a public repository. For example, in addition to summary statistics, the data points behind means, medians and variance measures should be available. If there are restrictions on publicly sharing data—e.g. participant privacy or use of data from a third party—those must be specified.

Reviewer #1: Yes

Reviewer #2: Yes

4. Is the manuscript presented in an intelligible fashion and written in standard English?

PLOS Global Public Health does not copyedit accepted manuscripts, so the language in submitted articles must be clear, correct, and unambiguous. Any typographical or grammatical errors should be corrected at revision, so please note any specific errors here.

Reviewer #1: Yes

Reviewer #2: Yes

5. Review Comments to the Author

Please use the space provided to explain your answers to the questions above. You may also include additional comments for the author, including concerns about dual publication, research ethics, or publication ethics. (Please upload your review as an attachment if it exceeds 20,000 characters)

Reviewer #1: Great Job to the team for the progress made so far!
Just a few comments, observations and suggestions for your consideration.
1, It would have been good to tie this study to its relevance in the public health spaces in terms of real life existing problems it seeks to solve or gaps to be addressed. e.g " to understand the experiences of 12 HCWs in the North West region of Cameroon with armed groups; evaluate how it affects healthcare delivery from HCWs perspective and examine HCWs coping mechanisms during the conflict with a view to informing health worker protection policies in conflict zones". You alluded to the need to better enforce the IHR implementation in your conclusion, which is good. I think this also needs to be reflected in the abstract and Introduction sections

Thanks for the positive comments. I agree it is important to mention the study’s relevance in Public Health. We were more focused on exploring the experiences of HCWs. We considered your comments and have made the necessary adjustments.

2,The Sample size could have been better more. 12 HCW in one district and mostly from one facility seems to me not very representative for the conclusions drawn.

We also thought 12 is not representative enough for an entire region. We would like to correct a few mistakes. The study was in one health facility and not the entire Health District. To improve the write-up, we rephrased the title which now reads: ‘Conflict, Healthcare and Professional Perseverance: A Qualitative Study in a Remote Hospital in an Anglophone region of Cameroon.

We also considered the principle of saturation in order not to be redundant (see methodology under data section – last but one paragraph)

3, The survey tool could have been richer if there was a segment to sought to understand specific changes or actions respondents would have desired. This is not a major limitation however.

We will consider this in subsequent studies

Reviewer #2: The research is technically weak, lacks detail in several areas, and the rigor required for qualitative research.
Introduction – the authors must provide a bit more detail of what the Anglophone crisis in Cameroon is. Readers must get all the details about the crisis from this article without resorting to an internet search to find out what this crisis is about.
Setting – It is not clear whether “Batibo Health District” is a health facility (hospital), or an administrative district with multiple health facilities. While this becomes somewhat clear that the health district may mean a district hospital, this must be clarified since “Health District” can have many meanings depending on country.

Thanks for the sound remark. This has been clarified in the manuscript

Participants – The study details how participants were recruited from the “district” based on a criterion yet in the results, it becomes somewhat apparent that these healthcare workers all worked at one rural hospital. The authors should provide a breakdown (percentages) of the various categories of healthcare workers at the hospital (nurses, doctors, lab scientists) as these were the only ones recruited for the study, to justify the claim that indeed care was taken to have each group fairly represented.

See response to Reviewer 1 comment #2. A table has been provided showing the different HCW categories at the facility.

Data collection – this section is lacking in detail. It is not clear how many sessions were conducted for the FDGs. The authors attempt to explain data collection for both FDGs and interviews in the same sentences, leaving the reader unclear whether the entire data collection for the study was done in just over an hour.

This is pertinent. Clarifications have been made in the manuscript
