# Supplementary material for: Conflict, healthcare and professional perseverance: A qualitative study in a remote hospital in an Anglophone Region of Cameroon
Source: PLOS Glob Public Health. 2022 Nov 29;2(11):e0001145. doi: 10.1371/journal.pgph.0001145 (PMC10021219; doi:10.1371/journal.pgph.0001145)
Supplement: Attachment — Submitted filename: Response to Reviewers.docx [file pgph.0001145.s013.docx]

Juste Niba

19^th^ October 2022

Douala, Cameroon

The Editor,

PLOS Global Public Health

Dear Sir/Madam,

Rebuttal Letter

We recently submitted our manuscript entitled: Conflict, Healthcare and Professional Perseverance: A Qualitative study in a Remote Hospital in an Anglophone Region of Cameroon. You have asked for a revision of the manuscript. Please find below the comments regarding the issues you wanted us to address. Our added comments appear in red.

Yours sincerely,

Juste Niba

PGPH-D-22-01049R1
Conflict, Healthcare and Professional Perseverance: A Qualitative Study in a Remote Hospital in an Anglophone Region of Cameroon
PLOS Global Public Health

Dear Dr. Niba,

Thank you for submitting your manuscript to PLOS Global Public Health.

Please pardon my "minor revision" recommendation. You have addressed the concerns that the reviewers had. In some instances these have been addressed a little too well!

My recommendation would be to cut some of the changes that you have made and still address the reviewers comments -

1) The second paragraph of the Introduction: I recommend you delete "Over the years, it appeared that the autonomy and identity of the Southern Cameroonians (today’s NW and SW regions) was gradually being suppressed in an attempt by their French counterparts to have absolute control. Awareness of this stirred grievances and complaints which were apparently not well handled by the current regime." and "eventually a problem which seemed so small "

Thank you for the words of appreciation. The changes have been made

2) The fourth paragraph of the introduction: I recommend you move the sentence "The ultimate goal is to provide local authorities and stake holders with comprehensive evidence that will allow them make informed decisions concerning the welfare of HCWs in conflict settings." to after the three questions. I think the sentence is important; it reads better after the three questions.

Changes have been made as well
